# Zinc-induced cardiomyocyte relaxation in a rat model of hyperglycemia is independent of myosin isoform

**DOI:** 10.1186/1475-2840-11-135

**Published:** 2012-11-02

**Authors:** Ting Yi, Yaser Cheema, Sarah M Tremble, Stephen P Bell, Zengyi Chen, Meenakumari Subramanian, Martin M LeWinter, Peter VanBuren, Bradley M Palmer

**Affiliations:** 1 Department of Molecular Physiology and Biophysics, University of Vermont, 122 HSRF Beaumont Ave, Burlington, VT 05405, USA; 2 Department of Medicine, University of Vermont, Burlington, VT 05405, USA

**Keywords:** Streptozotocin, PTU, Type 1 diabetes, Cardiac, Iron, Sulfur

## Abstract

It has been reported previously that diabetic cardiomyopathy can be inhibited or reverted with chronic zinc supplementation. In the current study, we hypothesized that total cardiac calcium and zinc content is altered in early onset diabetes mellitus characterized in part as hyperglycemia (HG) and that exposure of zinc ion (Zn^2+^) to isolated cardiomyocytes would enhance contraction-relaxation function in HG more so than in nonHG controls. To better control for differential cardiac myosin isoform expression as occurs in rodents after β-islet cell necrosis, hypothyroidism was induced in 16 rats resulting in 100% β-myosin heavy chain expression in the heart. β-Islet cell necrosis was induced in half of the rats by streptozocin administration. After 6 wks of HG, both HG and nonHG controls rats demonstrated similar myofilament performance measured as thin filament calcium sensitivity, native thin filament velocity in the myosin motility assay and contractile velocity and power. Extracellular Zn^2+^ reduced cardiomyocyte contractile function in both groups, but enhanced relaxation function significantly in the HG group compared to controls. Most notably, a reduction in diastolic sarcomere length with increasing pacing frequencies, i.e., incomplete relaxation, was more pronounced in the HG compared to controls, but was normalized with extracellular Zn^2+^ application. This is a novel finding implicating that the detrimental effect of HG on cardiomyocyte Ca^2+^ regulation can be amelioration by Zn^2+^. Among the many post-translational modifications examined, only phosphorylation of ryanodine receptor (RyR) at S-2808 was significantly higher in HG compared to nonHG. We did not find in our hypothyroid rats any differentiating effects of HG on myofibrillar protein phosphorylation, lysine acetylation, O-linked N-acetylglucosamine and advanced glycated end-products, which are often implicated as complicating factors in cardiac performance due to HG. Our results suggest that the relaxing effects of Zn^2+^ on cardiomyocyte function are more pronounced in the HG state due an insulin-dependent effect of enhancing removal of cytosolic Ca^2+^ via SERCA2a or NCX or by reducing Ca^2+^ influx via L-type channel or Ca^2+^ leak through the RyR. Investigations into the effects of Zn^2+^ on these mechanisms are now underway.

## Background

The term diabetic cardiomyopathy refers to a state of cardiac dysfunction independent of associated coronary artery disease that arises within weeks of hyperglycemia (HG) leading to longer term diabetes mellitus (DM) [1]. Diabetic cardiomyopathy in humans and animals is characterized in part by elevated end-diastolic left ventricular (LV) pressure, reduced end-diastolic LV volume, impaired augmentation of LV function in response to physiological stresses, reduced phosphocreatine resources and reduced LV filling rates [1-5]. Patients with diabetic cardiomyopathy often experience exertional dyspnea and a higher incidence of hospitalization due to heart failure. Currently there are no proven effective treatments for long term management of diabetic cardiomyopathy.

Cardiomyopathic effects in rodents or rabbits with HG or type 1 DM induced by treatments targeting pancreatic β-islet cells [6,7] are characterized at the cardiomyocyte level by depressed adrenergic responsiveness [8,9], prolonged action potential duration [10,11], reduced or delayed calcium influx through sarcolemmal L-type channels [10], reduced SERCA2a content and calcium reuptake rate [11,12], greater calcium ion (Ca^2+^) leak through the sarcoplasmic reticulum (SR) release channels also called ryanodine receptors (RyR) [13-15], elevated cytosolic Ca^2+^ in diastole [15], and reduced function yet elevated content of mitochondria [16]. These and other findings indicate a broad spectrum of cellular and molecular mechanisms that underlie the elevated morbidity and mortality associated with diabetic cardiomyopathy [1,2].

The development of diabetic cardiomyopathy after 6 months of DM in mice is inhibited or reversed by the chronic administration of zinc [17]. While the cellular and molecular mechanisms responsible for zinc-induced protection against diabetic cardiomyopathy are not fully understood, there is evidence that the zinc-binding protein metallothionein (MT) plays a role in protection against oxidative stress associated with diabetic cardiomyopathy [17-20]. While MT expression may be important for long term protection against oxidative stress, zinc also appears protective against oxidative stress in the short term such as in ischemia-reperfusion injury [21,22]. How zinc provides MT-independent cardioprotection is not well understood. It is known that extracellular zinc ion (Zn^2+^) competes with Ca^2+^ for the cardiomyocyte L-type calcium channel [23,24]. In addition, release of SR Zn^2+^ through the ryanodine receptors (RyR) also appears to be regulated similarly to that of SR Ca^2+^[25]. Thus, the balance of calcium and zinc regulation would be a logical focus of inquiry in determining the underling mechanisms responsible for the protective effects of zinc during the development of diabetic cardiomyopathy.

Hypothyroidism accompanies experimental HG and DM and leads to the upregulation of cardiac β-myosin heavy chain (MyHC) in rodents [26-29]. The slow β-MyHC isoform can significantly diminish cardiac contraction-relaxation function compared to controls, which normally expressing the faster α-MyHC isoform [5,30-32]. Thus, this and other secondary effects of HG and DM on diminished thyroid status must not be erroneously attributed to primary insulin-dependent effects [26].

In the present study, we hypothesized that extracellular zinc ameliorates cardiomyocyte dysfunction symptomatic of the development of diabetic cardiomyopathy in rats with HG. To obviate complications of variable MyHC isoform expression after β-islet necrosis [29], we induced HG in hypothyroid rats expressing 100% β-MyHC. We found that cardiomyocyte contraction function was enhanced by HG, but relaxation function was reduced by HG. Exposure to extracellular Zn^2+^ reduced contraction function in both HG and nonHG controls, but enhanced relaxation function only in the HG. Our findings are consistent with intracellular Ca^2+^ overload with HG and DM at the cellular level that can be reduced by exposure to Zn^2+^.

## Methods

### Rat model

All procedures were reviewed and approved by the Institutional Animal Care and Use Committee of The University of Vermont College of Medicine and complied with the *Guide for the Use and Care of Laboratory Animals* published by the National Institutes of Health. Sixteen male Wistar rats (Charles River Laboratories) were fed an iodine-deficient 0.15% 6-n-propyl-2-thiouracil (PTU) diet (Harlan Teklan) for at least 12 weeks. After 6 weeks on the diet, eight rats received tail vein injection of 50 mg/g STZ prepared in sodium citrate solution. The remaining rats received injection of similar volume of vehicle. At least 6 weeks after induction of HG by STZ, rats were anesthetized with 1.5-3% inhaled isoflurane, and short-axis view M-mode echocardiography was performed using Acuson Sequoia C256 (Seimens Medical Solutions) fitted with a 15 MHz probe suitable for resolving dimensions of the rat heart. Hearts were removed, and subjects died of exsanguination. Papillary muscles were then taken for myofilament performance assessment, apexes were harvested for elemental analysis and cardiomyocytes were isolated from the remaining LV.

### Echocardiography

Measures of heart size and function included heart rate, left ventricular posterior wall thickness (LVPWT), left ventricular diameter at end-diastole (LVD dias) and end-systole (LVD sys), fractional shortening = (LVD dias - LVD sys)/LVD dias, ejection fraction = (LVD dias^3^ - LVD sys^3^)/LVD dias^3^, ejection time taken between QRS complex and peak shortening, and circumferential velocity = fractional shortening/ejection time.

### Solutions for skinned myocardial strips

Solution concentrations (mmol/L) were formulated by solving equations describing ionic equilibria [33]. Relaxing solution: pCa 8.0, 5 EGTA, 5 MgATP, 1 Mg^2+^, 35 phosphocreatine (PCr), 300 U/mL creatine kinase (CK), ionic strength 200, pH 7.0. Activation solution: same as relaxing with pCa 4.0. Storage solution: same as relaxing with 10 μg/mL leupeptin and 50% glycerol wt/vol.

### Skinned myocardial strips

Skinned myocardial strips were studied as previously described [34]. Papillary muscles from the LV were dissected to yield at least two thin strips (~140 μm diameter, ~800 μm length) with longitudinally oriented parallel fibers, skinned for 2 hr at 22°C, and stored at −20°C. Aluminum T-clips were attached to the ends of a strip ~300 μm apart. The strip was mounted between a piezoelectric motor (Physik Instrumente, Auburn, MA) and a strain gauge (SensorNor, Horten, Norway), lowered into a 30 μL droplet of relaxing solution (pCa 8.0) maintained at 37°C, and incrementally stretched to 2.2 μm sarcomere length measured by digital Fourier Transform (IonOptix Corp, Milton, MA). Strips were calcium activated from pCa 8.0 to pCa 4.8.

Recorded forces were normalized to cross-sectional area to provide isometric tension (T). Individual recordings of T minus relaxed tension (T_min_) were then normalized to maximum developed tension (T_max_–T_min_) and fit to the Hill equation:

(1) $$\begin{array}{l} \left(\text{T}-{\text{T}}_{\text{min}}\right)/\left({\text{T}}_{\text{max}}–{\text{T}}_{\text{min}}\right)\\ \;={\left[{\text{Ca}}^{2+}\right]}^{\text{n}}/\left({{\left[{\text{Ca}}^{2+}\right]}_{50}}^{\text{n}}+{\left[{\text{Ca}}^{2+}\right]}^{\text{n}}\right)\text{,} \end{array}$$

where [Ca^2+^]_50_ = calcium concentration at half activation, pCa_50_ = −log [Ca^2+^]_50_, and n = Hill coefficient using a nonlinear least squares algorithm (Sigma Plot 8.0, SPSS, Chicago, IL).

### Tension-velocity relationship

The force-clamp technique was applied at maximal calcium activation. Various mechanical loads were expressed as a fraction of maximum absolute tension (T_max_). Force was maintained constant over a designated period of time using software-based feedback control, and the length change was continuously monitored. The tension-velocity (T-V) relationship was generated from these data.

The relationship was fit to a hyperbolic Hill equation normalized to T_max_:

(2) T'+a'V+b=1+a'b,

where T'= T/T_max_, a'= a/T_max_, and a and b are the parameters of the non-normalized hyperbolic Hill equation using a nonlinear least squares algorithm (Sigma Plot 8.0, SPSS, Chicago, IL). The physiological characteristics maximum unloaded shortening velocity (V_max_), velocity at maximum power (V_opt_), tension at maximum power (T`_opt_) and maximum power production (P_max_) were calculated from a' and b, as follows [31,32]:

(3) Vmax=b/a'ML/sVopt=b1+1/a'1/2–a'ML/sT'opt=a'2+a'1/2–a'fraction of TmaxPmax=(1−T'opt)bT'opt/(a'+T'opt)fraction of Tmax.ML/s,

### Native thin filament isolation

Intact native thin filaments were isolated from ventricular tissue as described previously [35]. In brief, myofibrils were isolated from flash frozen muscle tissue and subsequently homogenized in the following solution (in mmol/L): 100 NaCl, 5 MgCl_2,_ 1 NaN_3,_ 1 ethylene glycol tetraacetic acid (EGTA), 5 MgATP, 10 Na_3_PO_4_ and 2 μg/ml leupeptin at a ratio 40 ml/gm of myofibril (pH 6.5). Debris and thick filament were pelleted with centrifugation 137,000 × g for 20 minutes. Native thin filaments were then collected with centrifugation (137,000 x g for 180 minutes) with the pellet being raised in low salt buffer (in mmol/L): 25 KCl, 25 imidazole, 1 EGTA, 5 MgCl_2_, 10 DTT. Protein concentration was determined for native thin filaments with a protein assay (Bio-Rad; Hercules, CA, USA) using bovine serum albumin as the standard. Chicken skeletal myosin was isolated as previously described. Chicken skeletal myosin was used as the motility substrate in the *in vitro* motility assay due to its temporal stability, creating consistency between experiments [35]. Native thin filaments were labeled with rhodamine-phalloidin (1:1 molar ratio) prior to use in the motility assay.

### In vitro motility assay

Native thin filament contractile performance was assessed using the *in vitro* motility as described previously [36]. In brief, myosin (100 μg/ml, unless otherwise noted) was applied for one minute to a nitrocellulose coated coverslip in a high salt buffer (in mmol/L): 300 KCl, 25 imidazole, 5 MgCl_2_, 2 EGTA and 10 DTT. The surface was then washed with bovine serum albumin (0.5 mg/ml) in low salt buffer. Employing epifluorescent microscopy, rhodamine-labeled thin filaments were observed moving across the myosin coated surface in the presence of MgATP (2 mmol/L) in a buffered motility solution (in mmol/L unless otherwise noted): 25 KCl, 25 imidazole, 5 MgCl_2_, 2 EGTA and 10 DTT, glucose oxidase 0.1 mg/ml, catalase 1.8 μg/ml, glucose 2.3 mg/ml, and 0.38% methylcellulose. Free calcium was varied in the motility solution (pCa 10–4.0). Thin filament motility was recorded on videotape, and subsequently analyzed with the motion analysis system VP110 (Motion Analysis Corporation, Santa Rosa, CA). Typically >300 individual filament velocities were averaged to determine the mean velocity of each experiment. All motility experiments were performed at 30°C.

### Isolated cardiomyocyte function

Rat LV cardiomyocytes were isolated using retrograde perfusion of collagenase solution according to methods described elsewhere [37]. Cardiomyocytes were observed with a 40× objective on an inverted microscope (Nikon Diaphot) while bathed in normal Tyrode’s solution containing in mmol/L: 137 NaCl, 5.4 KCl, 1.2 CaCl_2_, 0.5 MgCl_2_, 10 HEPES, 5.5 glucose, 0.5 probenecid at 37°C (pH 7.4). Cardiomyocytes were electrically paced at 2, 4 and 6 Hz for at least 5 minutes at each frequency. Half of the cardiomyocytes were exposed to Tyrode’s with 32 μM Zn-acetate. Dynamic sarcomere length was detected by Fourier Transform of the digital image.

A subset of cardiomyocytes from each heart was loaded with Fura-2 AM (Invitrogen, Carlsbad, CA) and excited at 365 nm and 400 nm. Emission at 510 nm was used to detect Fura-2 fluorescence at each pacing frequency, although not with zinc exposure. We did not measure intracellular Ca^2+^ during Zn^2+^ exposure, because Fura-2 is sensitive to both physiological Zn^2+^ and Ca^2+^[23] and we would not have been able to differentiate the two ions. Background fluorescence was detected after lysing cardiomyocytes with 4 μM digitonin. Parameters of fluorescence ratio 365/400 (R) dynamics were used to represent those of calcium dynamics [38].

### Myosin isoform determination and western blots

Ventricular myosin isoform content of HG and nonHG rats was determined by the method of Reiser and Kline [39] with the use of Fluormax-2 Imaging analysis (Bio-Rad, Hercules, CA). Protein phosphorylation stain (Pro-Q diamond, Invitrogen) and Western blots for myosin regulatory light chain (MLC2) content (cat. ab92721, Abcam, San Francisco, CA), MLC2 phosphorylation (MLC2-P) at Serine-19 (cat. ab2480, Abcam), RyR (cat. MA3-916, Thermo Scientific), RYR phosphorylation at Serine-2808/9 (cat. A010-30AP, Badrilla), O-linked N-Acetylglucosamine (cat. ab2739, Abcam), lysine acetylation (cat. #9441, Cell Signaling Technologies) and pentosidine (PEN012, Biologo) were performed using tissue homogenates prepared in (mmol/L) 300 KCl, 25 Imidazole, 5 MgCl_2_, 2 EGTA, 10 DTT and pH 7.4. In some cases, myofilament fraction was extracted as the pellet after centrifugation and non-myofilament fraction as the supernatant.

### Cardiac element content measurements

Polypropylene conical tubes of 15 mL volume were soaked in 0.4 mM EGTA overnight, then rinsed four times with ddH_2_O and allowed to drip dry according to published recommendations [40]. Between 40–80 mg of each LV sample was placed in its own tube and lyophilized by 90 min of vacuum freeze drying. Samples were digested in 10 μL nitric acid (15.8 Normal) per mg tissue wet weight. Gallium was added in the amount 0.2 μL of 1 g/L Ga standard per mg tissue wet weight. Finally, ddH2O was added to bring total volume to 200× dilution of original tissue wet weight including final concentrations of 0.8 N nitric acid and 1 ppm Ga. Standards were prepared for elements Ca, Cu, Fe, K, Mg, Na, P, S and Zn over ranges 0–0.4 ppm, 0–4 ppm or 0–40 ppm depending on the element and all including 0.8 N nitric acid and 1 ppm Ga. Detection of element content was performed using inductively coupled plasma atomic emission spectroscopy (ICP-AES) performed by the ULTIMA2C operated in Poly-mode (Horiba Scientific, Edison, NJ). Element content was detected in triplicate for each sample and normalized against the Ga internal standard to provide a measure of element content in ppm relative to tissue wet weight.

### mRNA analysis and quantitative PCR

Total RNA was extracted from the rat hearts using Qiagen RNeasy kit and cDNA was synthesized using Invitrogen Superscript III first-strand synthesis system according to manufacturer instructions. Taqman primer sets were purchased from Applied Biosystems (Life Technologies): reference numbers HPRT Rn01527840, MT1a Rn00821759, ZnT2 Rn00563633 and γ-GCSh Rn00689046. Cycle threshold (Ct) values from quantitative RT-PCR were obtained from ABI Prism 7900HT sequence detection system. The mRNA expression levels measured as Ct were normalized to that of the endogenous control, HPRT, as ΔCt. The average ΔCt of the nonHG controls was used as the normalization factor to determine ΔΔCt for the HG group. Relative fold difference (RQ) in transcription levels between the HG and nonHG groups was calculated as RQ = 2^-ΔΔCt^.

### Statistical analysis

Analysis was performed using PASW Statistics 18.0 (SPSS). Multiple measurements of myofilament performance from the same heart were averaged to provide a single measure for that heart. Tension and actin velocity were normalized to values at maximal calcium activation and fit to the Hill equation using a non-linear least squares algorithm (Sigma Plot 8.0, SPSS, Chicago, IL). Repeated-measures ANOVA, using stimulation frequency within the same cardiomyocyte and zinc exposure within the same heart as repeated measures, was used to detect the relative effects of frequency and zinc exposure between HG and nonHG controls. Data are presented as mean ± SEM. For function analyses, n = 8 in each group. Significance by statistical tests are reported at the *P*<0.05, *P*<0.01 and *P*<0.001 levels. Trends at *P*<0.1 are also reported if in support of other statistically significant results.

## Results

### Animal characteristics

STZ induced a dramatic rise in serum glucose concentration in the HG rats, indicative of depressed insulin production by the pancreas (Table 1). Other notable consequences of 6 wks of HG included a reduction in body weight. There were no other highly significant effects of HG in the context of hypothyroidism on cardiac dimension or performance as measured by echocardiography. There was a trend (*P*<0.10) for a prolonged ejection time with HG, which reflects a prolonged activation of the force-producing myofilaments at the end of systole.

**Table 1:** Rat model characteristics, echo morphometry and serum glucose

	**HG (n=8)**	**nonHG (8)**
Age (wks)	19.8 ± 0.3	20.1 ± 0.4
PTU diet (wks)	12.8 ± 0.3	13.1 ± 0.4
Body mass (g)	217 ± 5 †	278 ± 15
Glucose 1 wks (mg/dL)	287 ± 20	no measure
Glucose 6 wks (mg/dL)	422 ± 21 ††	75 ± 6
Heart rate (bpm)	213.3 ± 9.1	229.1 ± 5.0
LVD dias (mm)	6.99 ± 0.18	6.88 ± 0.26
LVD sys (mm)	4.40 ± 0.16	4.41 ± 0.25
Fractional Shortening (%)	37.0 ± 1.7	35.9 ± 2.3
Ejection Fraction (%)	74.7 ± 1.9	73.0 ± 2.7
Ejection Time (ms)	146.6 ± 5.6 #	135.8 ± 2.7
Circum. Velocity (circ/s)	2.56 ± 0.17	2.67 ± 0.21
LVPWT dias (mm)	1.35 ± 0.09	1.23 ± 0.09

Statistical differences by two-tailed *t*-test between HG and nonHG. #*P*<0.10, †*P*<0.01, ††*P*<0.001.

### Myofilament performance

**Figure 1:**
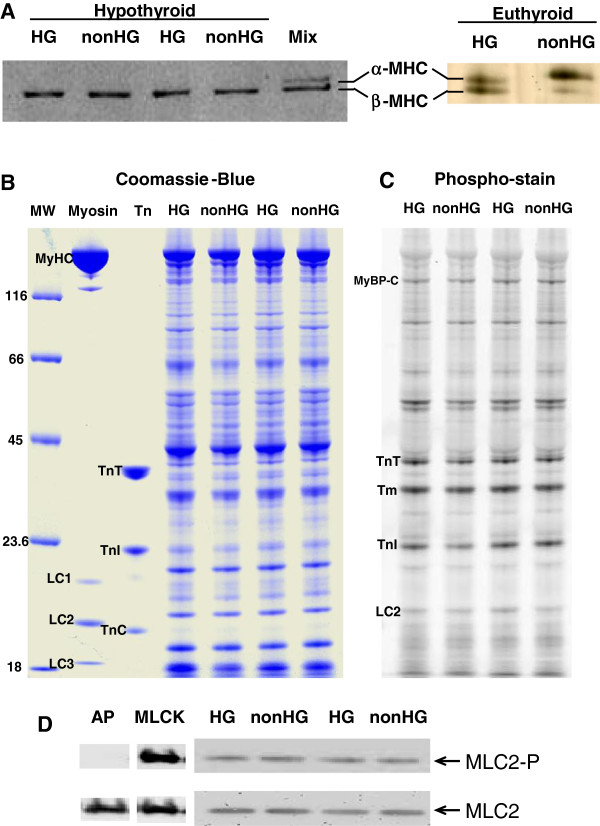
**Myosin isoforms and myofibrillar protein phosphorylation.A**. Gel electrophoresis stained with Coomassie-Blue demonstrate 100% β-MyHC content in both HG and nonHG groups due to hypothyroidism. In euthyroid rats, HG results in a significant upregulation of cardiac β-MyHC compared to the α-MyHC predominately expressed in the nonHG rat heart. **B **and **C**. Coomassie-blue and phospho-stain by Pro-Q Diamond indicated similar phosphorylation profiles of myofibrillar proteins in HG and nonHG. **D**. Western blots of phosphorylated MLC2 (MLC2-P) and total MLC content demonstrated similar degrees of phosphorylation in HG and nonHG controls. Additional lanes indicating alkaline phosphatase (AP) treatment to dephosphorylate MLC2 and myosin light chain kinase (MLCK) treatment to phosphorylate MLC2 provided negative and positive controls for MLC2 phosphorylation. Myosin = chicken skeletal myosin standard, Tn = bovine cardiac troponin standard, MyBP-C = myosin binding protein-C, Tm = tropomyosin, LC = myosin light chain.

Hypothyroidism inhibited the expression of α-MyHC and resulted in 100% β-MyHC incorporation into both HG and nonHG rats as shown in Figure 1A. In euthyroid rats, a similar bout of HG due to STZ resulted in a shift of the myosin isoform profile from a ~85/15 ratio of α-MyHC/β-MyHC in the nonHG to ~50/50 ratio in the HG (Figure 1A). Unless specified, we examined the hypothyroid rats and thus eliminated the complications of different myosin isoform mixtures [29]. Phosphorylation of myofibrillar proteins and specifically MLC2 were similar between HG and nonHG control rats (Figure 1B1D).

### Myofilament calcium sensitivity

**Figure 2:**
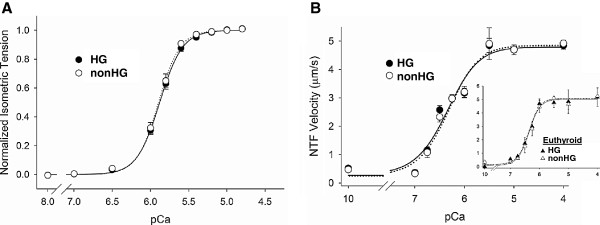
**Thin filament characteristics and myofilament performance.A**. Normalized isometric tension-calcium relationships of HG and nonHG controls were not different from each other, indicating similar thin filament sensitivities to calcium activation. **B**. Native thin filament (NTF) velocity-calcium relationships detected using the myosin motility assay were also similar between HG and nonHG demonstrating similar calcium sensitivity and myosin-actin MgATPase activity. Inset illustrates that HG did not affect NTF function in euthyroid rats not PTU-treated.

Minimum tension, maximal tension and calcium-activated developed tension of skinned myocardial strips were not different between HG and nonHG controls (Table 2). Calcium sensitivity of developed tension, visualized as the normalized tension-calcium relationships in Figure 2A, were indistinguishable between the HG and nonHG strips at all pCa conditions. The sensitivity of the thin filament to calcium activation, indicated by pCa_50_, and the cooperativity coefficient, n_Hill_, were also unchanged by HG (Table 2).

**Table 2:** Characteristics of myofilament force production in the skinned strip and of native thin filament (NTF) velocity in the myosin motility assay

	**HG (n=6)**	**nonHG (6)**
T_min_ (mN/mm^2^)	4.24 ± 0.33	4.87 ± 0.18
T_max_ (mN/mm^2^)	15.8 ± 1.4	16.9 ± 1.7
T_dev_ (mN/mm^2^)	11.6 ± 1.6	12.1 ± 1.8
pCa_50_	5.88 ± 0.02	5.89 ± 0.02
n_Hill_	3.41 ± 0.38	3.06 ± 0.16
	(n=3)	(3)
V_Ca, NTF_ (µm/s)	4.52 ± 0.30	4.62 ± 0.29
pCa_50, NTF_	6.35 ± 0.07	6.32 ± 0.06
n_Hill, NTF_	1.36 ± 0.22	1.39 ± 0.23

T_min_= minimum tensile stress, T_dev_ = developed tensile stress, pCa_50_ = pCa at 50% calcium activation, n_Hill_ = Hill coefficient, V_Ca, NTF_ = calcium-dependent NTF velocity.

The velocity-calcium relationships of the native thin filaments (NTF) isolated from the HG and nonHG control rats were indistinguishable from each other (Figure 2B). Parameters for maximal NTF calcium-activated velocity and calcium sensitivity of NTF were also similar in HG and nonHG (Table 2). Moreover, NTF function was not affected by STZ treatment in euthyroid rats (Figure 2B, inset). These results for tension-calcium and NTF velocity-calcium relationships indicate that thin filament function was not affected by 6 wks of HG in hypothyroid or euthyroid rats.

### Tension-velocity relationship

**Figure 3:**
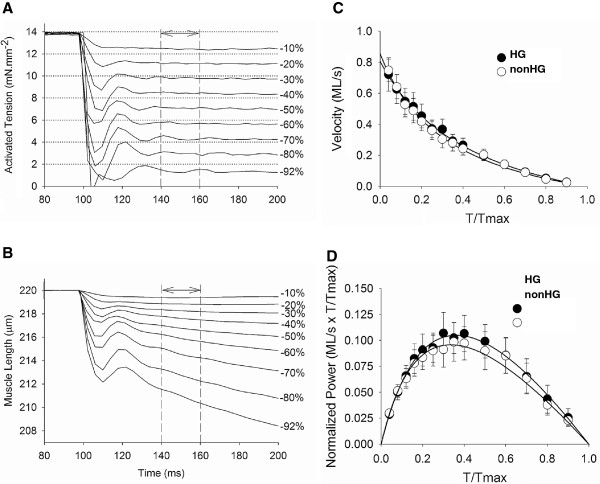
**Example of force-clamp technique and results of tension-velocity and tension-power relationships.A**. The force clamp technique relies upon using the force signal as feedback to the muscle length so that a preset force is achieved. The preset force varies from 10-96% and several examples are shown here. **B**. The resulting length change indicates velocity of contraction at the preset force. Velocity was calculated between 40–60 ms after the change in load. **C-D**. Tension-velocity and tension-power relationships were similar in HG and nonHG controls.

Examples of clamped force and corresponding length changes, which illustrate the force-clamp technique, are shown in Figures 3A and 3B. Velocity was calculated over 40–60 ms after force-clamp was initiated, which allowed for steady force maintenance and was always within 5% of the originally set sarcomere length. Tension-velocity and tension-power relationships recorded from force-clamp experiments are shown in Figures 3C and 3D. We found no differences in the velocity- and power-tension relationships between HG and nonHG controls. Parameters of the maximum velocity, optimum velocity, optimum tension and maximum normalized power production, which are indicators of myofilament contractile performance, were also not different between HG and nonHG controls (*P*>0.10, Table 3).

**Table 3:** Characteristics of force-velocity relationships in skinned strips

	**HG (n=6)**	**nonHG (6)**
a/T_max_ (no units)	0.501 ± 0.086	0.392 ± 0.102
b (10^-3^ ML/s)	414 ± 104	347 ± 95
V_max_ (10^-3^ ML/s)	817 ± 125	899 ± 85
V_opt_ (10^-3^ ML/s)	292 ± 45	291 ± 35
T_opt_/T_max_ (%)	35.8 ± 1.5	32.5 ± 2.8
P_max_ (10^-3^ ML/s × T/T_max_)	106 ± 18	98 ± 17

a/T_max_ = curvature index, b = velocity index, V_max_ = extrapolated maximum unloaded velocity, V_opt_ = velocity at maximum power, T_opt_/T_max_ = relative tension at maximum power, P_max_ = maximum normalized power.

### Cardiomyocyte sarcomere dynamics

**Figure 4:**
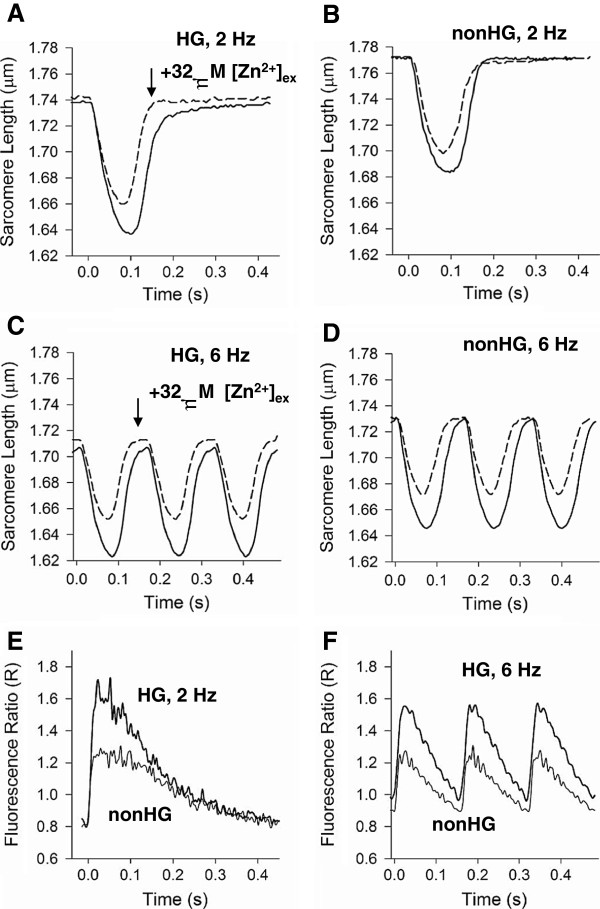
**Representative cardiomyocyte sarcomere shortening-relengthening transients with (−−-) and without (—) 32** **μM extracellular Zn**^**2+**^**and intracellular Ca**^**2+**^**transients.A **and **B**. At 2 Hz cardiomyocyte shortening was greater in HG compared to nonHG. Diastolic sarcomere length was noticeably shorter in HG compared to nonHG at 2 Hz. Extracellular Zn^2+^ reduced cardiomyocyte shortening in both HG and nonHG and led to longer diastolic sarcomere lengths in HG. **C **and **D**. At 6 Hz extracellular Zn^2+^ reduced cardiomyocyte shortening in both HG and nonHG. Diastolic sarcomere length was still noticeably shorter in HG compared to nonHG, and exposure to Zn^2+^ led to longer diastolic sarcomere lengths in the HG group. **E **and **F**. At 2 and 6 Hz, fluorescence ratio transients of HG and nonHG cardiomyocytes were similar in temporal characteristics, but there was a greater area under Fura-2 fluorescence ratio transient in the HG at 2 Hz and a trend at 6 Hz.

Representative cardiomyocyte sarcomere dynamics are illustrated in Figures 4A-D. Cardiomyocyte sarcomere shortening at 2 Hz (Figure 4A and 4B) was greater in the HG (6.46 ± 0.28%) compared to nonHG controls (5.48 ± 0.28%, *P*<0.05), but not at 6 Hz (Figures 4C and 4D). Diastolic sarcomere length at all pacing frequencies was shorter in the HG (1.736 ± 0.010 μm at 2 Hz) compared to nonHG controls (1.770 ± 0.007 μm at 2 Hz, *P*<0.05). In both groups, diastolic sarcomere length shortened as pacing frequency increased, i.e., there was a lack of full relaxation with increasing pacing frequency. The HG group displayed a greater lack of full relaxation with pacing frequency compared to nonHG controls.

Exposure to extracellular Zn^2+^ reduced sarcomere shortening in both HG and nonHG. Extracellular Zn^2+^ enhanced diastolic sarcomere length significantly in the HG, particularly as pacing frequency increased, but did not significantly affect diastolic sarcomere length in the nonHG. Quantitative analyses regarding the sensitivity of contraction-relaxation function to HG, pacing frequency and Zn^2+^ are presented below.

### Contraction function

**Figure 5:**
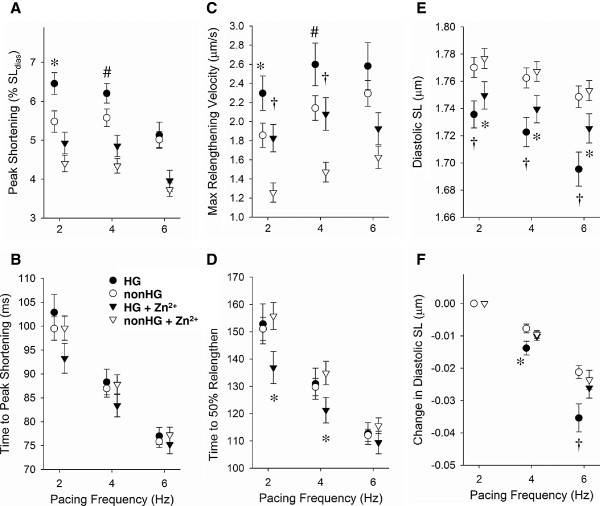
**Parameters of cardiomyocyte sarcomere dynamics.A**. Peak cardiomyocyte shortening was greater in HG compared to nonHG at 2 and 4 Hz, but not at 6 Hz. Exposure to 32 μM [Zn^2+^]_ex_ reduced peak shortening in both groups and abolished differences in peak shortening between the groups. **B**. Time to peak shortening was not different between HG and nonHG and not affected by zinc exposure. **C**. Both with and without [Zn^2+^]_ex_, maximum velocity of relengthening was greater in HG compared to nonHG at 2 and 4 Hz, but not at 6 Hz. Maximum velocity of relengthening in the nonHG group was enhanced with pacing frequency, but not as much in the HG group. **D**. Time to 50% relengthening was enhanced by 32 μM [Zn^2+^]_ex_ in the HG group only. **E**. Diastolic sarcomere length was shorter in HG compared to nonHG and shortened in both groups by increasing pacing frequency. **F**. The change in diastolic sarcomere length with pacing frequency was greatest in HG cardiomyocytes not exposed to [Zn^2+^]_ex_. Exposure to 32 μM [Zn^2+^]_ex_ in the HG cardiomyocytes recovered a normal response of diastolic sarcomere length to pacing frequency. #*P*<0.1, *P<0.05, †*P*<0.01; n=8 in each group.

The variables used here to represent cardiomyocyte contractile function were peak sarcomere shortening and maximum shortening velocity. Without Zn^2+^ exposure, cardiomyocyte peak shortening and maximum shortening velocity were statistically enhanced in HG compared to nonHG at 2 and 4 Hz pacing frequency, but not at 6 Hz (Figure 5A). Contractile function was reduced with increasing pacing frequency indicated by significant ‘freq’ main effects (*P*<0.001 in Table 4). There were also significant ‘HG×freq’ interactions for peak shortening and maximum shortening velocity (*P*<0.01 in Table 4) suggesting that the HG group was more susceptible to the reduction in contractile function with increasing pacing frequency. Exposure to extracellular Zn^2+^ reduced peak shortening and maximum shortening velocity (note significant ‘zinc main effect’ *P*<0.001 for these variables in Table 4) in both groups and subdued any differences between HG and nonHG controls (Figure 5A).

**Table 4:** Results of repeated measures ANOVA of cardiomyocyte shortening-relengthening characteristics

**Variable**	**HG**	**freq**	**HG×freq**	**zinc**	**HG×zinc**	**freq×zinc**	**HG×freq×zinc**
Peak Shortening (%)	0.013*	**<0.001**†	**0.008**†	**<0.001**†	0.941	0.619	0.424
Peak Shortening (μm)	0.043*	**<0.001**†	**0.008**†	**<0.001**†	0.992	0.698	0.374
Max Shortening dSL/dt (μm/s)	0.028*	<**0.001**†	0.016*	**<0.001**†	0.922	0.624	0.582
Max Lengthening dSL/dt (μm/s)	0.002*	**<0.001**†	0.011*	**<0.001**†	0.833	0.121	0.827
Time to Peak (ms)	0.712	**<0.001**†	0.295	0.354	0.158	0.153	0.674
Time to 10% return (ms)	0.505	**<0.001**†	0.082#	0.294	0.215	0.094#	0.706
Time to 50% return (ms)	0.230	**<0.001**†	**<0.001**†	0.554	0.249	0.368	0.534
Diastolic SL (μm)	**<0.001**†	**<0.001**†	**0.005**†	0.722	0.913	0.176	0.037*

The table presents *P*-values for the main effects of HG, freq and zinc and for interactions of HG×freq, HG×zinc, freq×zinc and HG×freq×zinc. #*P*<0.10, *P<0.05, †*P*<0.01; n=8 in each group.

### Relaxation function

The variables used here to represent cardiomyocyte relaxation function were maximum relengthening velocity, times to 50% relengthening, and diastolic sarcomere length. Without Zn^2+^ exposure, maximum relengthening velocity was significantly enhanced in the HG at 2 and 4 Hz, but not at 6 Hz (Figure 5C). Maximum relengthening velocity was consistently enhanced with pacing frequency in the nonHG controls (Figure 5C), but not as much in the HG group (note significant freq main effect *P*<0.001 and HG×freq interaction *P*=0.011 in Table 5). These latter results indicate a reduced relaxation-frequency response due to HG. During Zn^2+^ exposure, maximum relengthening velocity remained significantly enhanced in the HG group compared to nonHG controls at 2 and 4 Hz (Figure 5C).

**Table 5:** Results of repeated measures ANOVA of cardiomyocyte calcium transient characteristics

**Variable**	**HG**	**freq**	**HG×freq**
Peak – Diastolic Ratio (R)	0.107	0.250	0.828
Area of Ratio Transient (R.ms)	0.079#	**<0.001**†	0.179
Time to Peak (ms)	0.673	**<0.001**†	0.941
Time to 50% return (ms)	0.111	**<0.001**†	0.546
Exponential Time Constant (ms)	0.514	**<0.001**†	0.815
Diastolic Ratio (R)	0.340	0.100	0.532

The table presents *P*-values for the main effects of HG and freq and for interactions of HG×freq. #*P*<0.10, *P<0.05, †*P*<0.01; n=8 in each group.

Time to 50% relengthening (Figure 5D) was not different between HG and nonHG unless cardiomyocytes were exposed to Zn^2+^. Specifically, during Zn^2+^ exposure, time to 50% relaxation was significantly shorter in the HG compared to nonHG at 2 and 4 Hz, but not 6 Hz (Figure 5D). A similar effect, although not statistically significant (*P*<0.15), was observed for time to 10% relengthening (not shown) and time to peak (Figure 5B).

Diastolic sarcomere length was significantly shorter in the HG compared to nonHG at all pacing frequencies both without and during Zn^2+^ exposure (Figure 5E). Diastolic sarcomere length shortened with increasing pacing frequency in both HG and nonHG (note ‘freq’ main effect *P*<0.001 in Table 4), but significantly more so in the HG compared to nonHG (indicated by HG×freq interaction *P*=0.005 in Table 4).

As noted in Figure 5E, the reduction in diastolic sarcomere length with pacing frequency in the HG group appeared more subtle during Zn^2+^ exposure. Indeed, there was a HG×freq×zinc interaction *P*=0.037 for diastolic sarcomere length, which indicated a different sensitivity to pacing frequency between the groups that was dependent upon Zn^2+^ exposure. This interaction can be visualized more easily in Figure 5F, where the change in diastolic sarcomere length from that at 2 Hz is illustrated. As seen in Figure 5F, diastolic sarcomere length was reduced with increasing pacing frequency in both groups and significantly more so in the HG without Zn^2+^. The diastolic sarcomere length in the HG cardiomyocytes exposed to Zn^2+^ responded to pacing frequency similar to the nonHG group (Figure 5F).

In summary, relaxation function measured as maximum relengthening velocity, time to 50% relengthening and diastolic sarcomere length in the HG cardiomyocytes was more sensitive to the enhancing effects of extracellular Zn^2+^ compared to that in nonHG controls.

### Cardiomyocyte calcium dynamics

**Figure 6:**
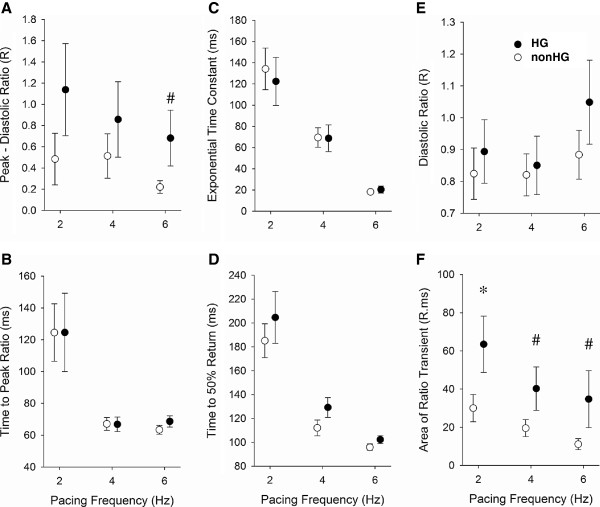
**Parameters of cardiomyocyte calcium dynamics.A**. Peak fluorescence ratio was not different between HG and nonHG, except at 6 Hz where there was a trend for higher peak ratio in HG. **B-E**. Time to peak fluorescence ratio, exponential decay time constant, time to 50% decay in fluorescence ratio and diastolic fluorescence ratio were not different between HG and nonHG. **F**. Area under the fluorescence ratio transient was higher in HG compared to nonHG at 2 Hz and demonstrated similar trend at 4 and 6 Hz. #*P*<0.1, *P<0.05; n=8 in each group.

Figures 4E and 4 F illustrate representative fluorescence ratio transients that reflect calcium dynamics in the cardiomyocytes of the HG and nonHG groups not exposed to Zn^2+^. Indices of systolic and diastolic performance are presented in Figure 6. The developed peak fluorescence ratio (R), i.e., peak minus diastolic R (Figure 6A), was not statistically different between HG and nonHG, although we detected a trend for higher developed peak R at 6 Hz. Time to peak R, exponential time constant for R decay, time to 50% R decay and diastolic R were not different between HG and nonHG at any pacing frequency (Figures 6B-6E). Contrary to the higher SERCA2a expression and SR Ca^2+^ reuptake rate reported for rats with type 2 DM [41], we did not detect a greater SR Ca^2+^ reuptake rate in the HG cardiomyocytes. However, the area under the R transient, not including the area under diastolic R, was greater in the HG compared to nonHG at all pacing frequencies (Figure 6F and Table 5).

These data would suggest that there was greater total cytosolic calcium over each cardiac cycle in the HG compared to nonHG in the absence of Zn^2+^ exposure. A greater total cytosolic calcium in the HG could explain in part the enhanced contraction function in the HG at the 2 Hz and 4 Hz pacing frequencies.

### Other post-translational modifications

**Figure 7:**
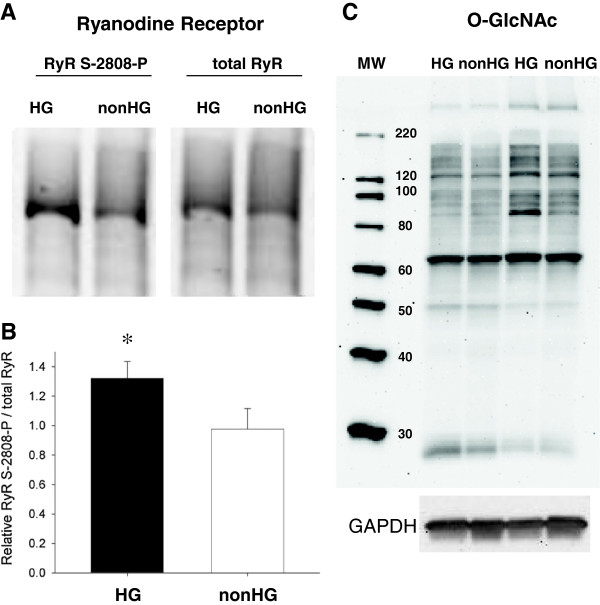
**Post translational modifications due to HG.A and B**. RyR phosphorylation at S-2808 normalized to total RyR content was significantly enhanced in HG compared to nonHG. **C.** O-linked N-Acetylglucosamine (O-GlcNAc) was not apparent in myofibrillar proteins except possibly an interfilament protein at ~65 kDa, which was not differentially affected by HG. MW = molecular weight marker. *P<0.05; n=4.

We examined several post-translational modifications implicated as significant modifiers of cardiomyocyte function in HG. We found RyR phosphorylation at S-2808 was enhanced in HG compared to nonHG (Figures 7A and 7B) as has been reported previously and implicated as a contributor of increased SR Ca^2+^ leak in HG [15]. The elevated phosphorylation of RyR S-2808 may result from a moderately elevated adrenergic environment in the myocardium due to reduced reuptake of norepinephrine in sympathetic nerves [42].

O-linked N-Acetylglucosamine (O-GlcNAc), which is known to occur with complications in HG and DM [43], was highly variable in both HG and nonHG samples, but was not detected on the major myofilament proteins (Figure 7C). The protein most subject to O-GlcNAc had a molecular weight of ~65 kDa, possibly one of the intermediate filament proteins synocoilin or desmin, but was not differently affected in HG and nonHG controls (Figure 7C).

**Figure 8:**
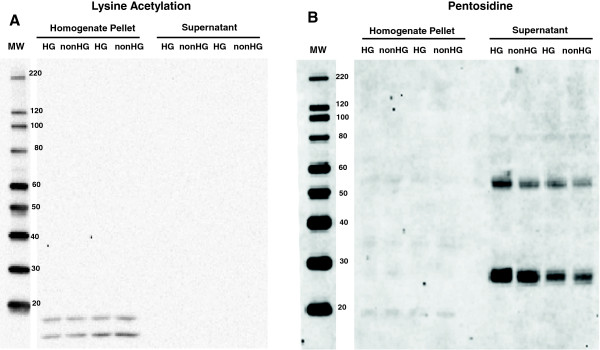
**Post translational modifications due to HG.** Myofilament (pellet) and non-myofilament (supernatant) proteins were separated by centrifugation of tissue homogenate. **A**. Lysine acetylation was not detected for any myofibrillar protein except two unidentified proteins at <20 kDa. These were not differentially acetylated between HG and nonHG. **B.** Advanced glycation end-products (AGEs), detected by Western blots of anti-pentosidine probe, demonstrated similarly low AGEs accumulation in myofilament fraction between HG and nonHG. AGEs accumulation of non-myofilament proteins appeared in two proteins at ~27 kDa and ~55 kDa, but was highly variable and not different between HG and nonHG.

Accumulation of advanced glycated end-products (AGE), implicated as a contributor to myocardial stiffness in HG [44], was detected using anti-pentosidine probe (Figure 8B). Neither the myofilament nor non-myofilament fractions of the tissue homogenate demonstrated differential AGE accumulation due to HG.

Lysine acetylation, which has been implicated as a significant modifier of myosin and myofilament function [45], was not apparent in myosin or in any of the major myofilament proteins (Figure 8A). Our examination of myofilament composition in terms of myosin isoform and of commonly assessed post-translational modifications of myofilament proteins did not result in our finding any significant differences between the HG and nonHG hypothyroid rats.

### Cardiac element content

HG in the hypothyroid rat led to elevations in iron and sulfur per cardiac tissue wet weight (Table 6), while there was no effect on the other elements. In particular, myocardial calcium and zinc concentrations were similar between the HG and nonHG hypothyroid rats. Euthryroid rats, however, responded to HG with an elevation in calcium, potassium, sodium and sulfur and a reduction in copper and iron (Table 6). Interestingly, neither the hypothyroid nor euthyroid rats demonstrated changes in magnesium, phosphorus or zinc content due to HG, and both hypothryroid and euthyroid rats demonstrated a ~20-25% increase in sulfur with HG.

**Table 6:** Cardiac element content and select gene expression at 6 wks after injection of STZ (HG) or vehicle (nonHG) in hypothyroid rats fed PTU diet or in euthyroid rats fed standard rat chow

	**Hypothyroid**		**Euthyroid**	
	HG (n=8)	nonHG (8)	HG (6)	nonHG (6)
Ca	101.2 ± 6.0	100.8 ± 7.4	284.4 ± 44.3*	155.4 ± 32.9
Cu	4.4 ± 0.2	3.9 ± 0.2	5.2 ± 0.3 *	6.4 ± 0.2
Fe	66.7 ± 3.0*	58.7 ± 1.9	95.5 ± 2.5 †	122.3 ± 7.5
K	2316 ± 148	2053 ± 85	7369 ± 793 *	5052 ± 566
Mg	186.8 ± 8.9	177.3 ± 7.6	275.1 ± 13.6	257.8 ± 6.0
Na	1863 ± 102	1788 ± 75	2325 ± 224 *	1641 ± 186
P	2158 ± 105	2012 ± 81	2726 ± 86	2711 ± 24
S	2545±155 *	2022 ± 102	3707 ± 163*	3149 ± 95
Zn	17.6 ± 0.7	17.7 ± 0.8	23.1 ± 1.1	22.2 ± 0.3
MT1a	-	-	0.66 ± 0.13 *	1.00 ± 0.05
ZnT2	-	-	0.76 ± 0.07 #	1.00 ± 0.10
γ-GCSh	-	-	1.30 ± 0.08 †	1.00 ± 0.03

Concentration given in ppm: μg of element per g wet weight of LV. Gene expression given in RQ = 2^-ΔΔCt^. normalized to HPRT expression and then to mean of nonHG. Differences between HG and nonHG controls reported at *P<0.05, †*P*<0.01.

### Gene expression

Zinc binds to the metal responsive element (MRE) on the promoter region of select genes and thereby signals activity of metal response element-binding transcription factor-1 (MTF-1) and the subsequent transcription of several genes related to zinc homeostasis and protection against oxidative stress: metallothionein-1 (MT1a), γ-glutamylcysteine synthase heavy chain (γ-GCSh) and zinc transporters-1 and -2 (ZnT1, ZnT2) [46-48]. Given the previous findings of an upregulation of MT1a in mouse hearts after STZ injection [19], we expected a similar upregulation of MTF-1 dependent genes in the hearts of our euthyroid HG rats. (We did not examine gene expression in the hypothyroid rats due to lack of tissue.) Instead we found that HG due to STZ injection led to a significant reduction in MT1a and ZnT2 expression, similar to a downregulation of MT1a in cardiac tissue of diabetic mouse embryos [49] (Table 6). Our data suggest that MTF-1 activity is reduced with HG, reflected in MT1a and ZnT2 downregulation, despite a normal level of Zn content. We also report an upregulation of γ-GCSh, which is consistent with the increased glutathione content in the hearts of diabetic mouse embryos [49]. The regulation of γ-GCSh in this case is likely driven by some other transcription factor other than MTF-1 [50], due in part to the promoter of the γ-GCSh gene bearing only a single MRE and may not be as dependent upon MTF-1 activity as MT and ZnT genes bearing 3–6 MRE’s [50].

## Discussion

We report here the effects of HG induced by β-islet necrosis on cardiomyocyte function in hypothyroid rats. Hypothyroidism was induced in these rats to ensure the expression of 100% β-MyHC in the ventricles of both disease and control groups. We found no significant effects of HG on echocardiographic measures of cardiac function or on myofilament performance in the context of hypothyroidism. These findings support the arguments raised by Dillmann [27], Davidoff et al. [5,26] and Rundell et al. [29] that modifications in myofilament function due to HG in rodents are primarily if not exclusively through myosin isoform shifts secondary to associated hypothyroidism. With our unique animal model of HG, we were able to demonstrate the effects of HG on cardiomyocyte function independent of differential MyHC isoform expression and its associated effects on myofilament performance and yet with greater relevance to the human heart, which expresses predominately β-MyHC.

### Cardiomyocyte Sensitivity to Zn^2+^ in HG

We found that extracellular Zn^2+^ enhanced relaxation function only in the cardiomyocytes of HG rats and not in the nonHG. Most notably, incomplete relaxation as pacing frequency increased was more prominent in HG, and extracellular Zn^2+^ exposure to the HG cardiomyocytes regained a normal relaxation response to pacing frequency. This is a novel and important finding that highlights the detrimental effect of HG on cardiomyocyte Ca^2+^ regulation and its amelioration by Zn^2+^. These results are consistent with the idea that HG leads to a higher cardiomyocyte intracellular Ca^2+^ load leading to incomplete relaxation and also a higher sensitivity to the relaxing effects of extracellular Zn^2+^.

The molecular mechanisms, which underlie cardiomyocyte relaxation affected by extracellular Zn^2+^ exposure or intracellular Zn^2+^ accumulation, may be targets for the treatment of diastolic dysfunction in diabetic cardiomyopathy. Due to the protean effects of zinc on cellular functions an exhaustive study of zincs effects on cardiomyocyte contractile function is beyond the scope of our current investigation. Intracellular Zn^2+^ is known to activate or deactivate a number of kinases and phosphatases, reviewed in Foster and Samman [51], as well as compete with Ca^2+^ for calcium regulatory proteins and channels [23,24]. Given the rapidity with which the extracellular Zn^2+^ exposure affected cardiomyocyte function and the recent findings that Zn^2+^ regulation appears to follow that of Ca^2+^[25], we would hypothesize that the most immediate effects of Zn^2+^ on cardiomyocyte function involve a competitive effect on Ca^2+^ regulatory mechanisms. By reducing the inward Ca^2+^ current through the L-type channel [23,24], for example, extracellular Zn^2+^ exposure could lower the open probability of RyR and presumably also reduce SR Ca^2+^ leak through the RyR, which has been shown to be elevated in HG [13,14]. Our finding an elevated RyR phosphorylation at S-2808 in our HG rats is consistent with that reported by others [15] and suggests that the hypothyroid state did not diminish the effects of HG on RyR post-translational modifications and possibly also its function. It must be noted, however, that HG-dependent enhancement of RyR open probability may be independent of S-2808 phosphorylation [14].

### Cardiomyocyte Ca^2+^ regulation in HG

HG in this model led to enhanced cardiomyocyte contraction function at lower than in vivo stimulation frequencies. These results suggest an elevated SR Ca^2+^ load and release in the HG cardiomyocytes, consistent with the greater area under the Fura-2 fluorescence ratio transient we observed in the HG. We also found a significantly reduced diastolic sarcomere length in the HG. The elevated cardiomyocyte contraction function and reduced diastolic sarcomere length in the absence of any variation in myofilament calcium sensitivity collectively suggest elevated systolic and diastolic intracellular Ca^2+^ concentrations with HG. However, we were not able to detect an elevated diastolic Ca^2+^ concentration with HG.

Our measures of intracellular Ca^2+^ by Fura-2 suggest that calcium regulation in the cardiomyocytes of the HG rat were altered to contribute to an elevated SR Ca^2+^ load at relatively low pacing frequencies (2–4 Hz). In the current study, we did not measure intracellular Ca^2+^ during exposure to extracellular Zn^2+^, because Fura-2 is sensitive to both physiological Zn^2+^ and Ca^2+^[23]. To our knowledge, there is no other Ca^2+^ dye sensitive to Ca^2+^ with a K_d_ < 1 μM (which would be necessary to detect intracellular Ca^2+^ dynamics), that is also insensitive to physiological intracellular Zn^2+^. Based on the response of sarcomere dynamics, we suspect that cardiomyocyte Ca^2+^ load, peak systolic Ca^2+^ and diastolic Ca^2+^ concentrations would be reduced as intracellular Zn^2+^ accumulated.

### Cardiac elemental content and gene expression

We are aware of only two other studies of the effects of β-islet necrosis on cardiac elemental content. LV contents of calcium and copper were reduced in the euthyroid rat after 6–8 wks HG due to STZ [52], and zinc is reduced in a mouse model after 6 wks HG due to alloxan [53]. Our findings in the euthyroid rat indicate significant changes in the regulation of several elements after HG induction by STZ; however, we would not be able to tell if these changes are due to the primary effects of HG or due to secondary effects of some other syndrome accompanying HG. Interestingly, gene expression levels for MT1a and ZnT2 were reduced in the euthyroid HG rats despite the normal levels of myocardial zinc. We speculate that HG nevertheless depresses activity of MTF-1, which is the common transcription factor to activate MT and ZnT2 [46-48]. We propose that this mechanism is reflected in the elevated sulfur content, which suggests a higher density of zinc-buffering sulfur-rich proteins like glutathione [49] and subsequently reduced zinc availability to signal MTF-1 activity [54].

In the hypothyroid rats, LV contents of iron and sulfur are elevated with 6 wks of HG and there were no other changes in elemental contents. Therefore, there was no zinc deficiency with HG that could account for the higher Zn^2+^ sensitivity in the HG cardiomyocytes. However, we speculate that an elevation in sulfur-rich proteins in the HG hearts reduces the zinc available to activate or deactivate kinases and phosphatases that affect phosphorylation status of calcium regulatory proteins such as RyR.

## Conclusions

We demonstrate here a unique and valuable animal model, the hypothyroid rat, which eliminated the complicating factors of disparate thyroid status, myofilament function, myosin isoform composition and post-translational modifications due to HG or DM in euthyroid animals. This model may prove particularly useful in further studies of the effects of HG and DM on cardiac cellular and molecular functions.

This report provides evidence supporting zinc administration as a possible long term management regimen for incomplete relaxation and diastolic dysfunction associated with diabetic cardiomyopathy. Human patients with type 2 DM demonstrate an increased mortality due to coronary heart disease (CHD) and myocardial infarction inversely proportional to serum zinc levels [55]. Thus, elevation of extracellular zinc may reduce the risks of CHD with DM, but also potentially enhanced diastolic function in these patients most at risk. The data presented here suggest that calcium regulatory mechanisms, particularly those responsible for cytosolic Ca^2+^ decline in diastole, are sensitive to Zn^2+^ exposure and more so sensitive in the diabetic heart despite normal total content of cardiac calcium and zinc. Further investigations into the Zn^2+^ sensitivity of calcium regulatory mechanisms responsible for relaxation, such as Na^+^-Ca^2+^ exchanger and SERCA2a, are warranted and currently underway.

## Competing interests

The authors declare that they have no competing interests.

## Authors' contributions

Experiments were conceived and designed by TY, YC, PV and BMP. Primary data were acquired by TY, YC, SMT, MS, SPB and ZC. Secondary analyses and interpretations were performed by TY, YC, MS, MML, PV and BMP. Manuscript and figures were drafted and proofed by TY, SMT, MML, PV and BMP. All authors read and approved the final manuscript.
